# Comprehensive analysis of hypoxia-related genes in diagnosis and immune infiltration in acute myocardial infarction: based on bulk and single-cell RNA sequencing data

**DOI:** 10.3389/fmolb.2024.1448705

**Published:** 2024-08-21

**Authors:** Guoqing Liu, Wang Liao, Xiangwen Lv, Miaomiao Zhu, Xingqing Long, Jian Xie

**Affiliations:** ^1^ Department of Cardiology, The First Affiliated Hospital of Guangxi Medical University, Nanning, Guangxi, China; ^2^ Department of Cardiology, The First People’s Hospital of Yulin, Yulin, Guangxi, China; ^3^ Department of Cardiology, The Second Affiliated Hospital of Guangxi Medical University, Nanning, Guangxi, China; ^4^ The First Affiliated Hospital of Guangxi Medical University, Nanning, Guangxi, China

**Keywords:** acute myocardial infarction, hypoxia, diagnostic model, single-cell analysis, immune infiltration

## Abstract

**Background:**

Hypoxia has been found to cause cellular dysfunction and cell death, which are essential mechanisms in the development of acute myocardial infarction (AMI). However, the impact of hypoxia-related genes (HRGs) on AMI remains uncertain.

**Methods:**

The training dataset GSE66360, validation dataset GSE48060, and scRNA dataset GSE163956 were downloaded from the GEO database. We identified hub HRGs in AMI using machine learning methods. A prediction model for AMI occurrence was constructed and validated based on the identified hub HRGs. Correlations between hub HRGs and immune cells were explored using ssGSEA analysis. Unsupervised consensus clustering analysis was used to identify robust molecular clusters associated with hypoxia. Single-cell analysis was used to determine the distribution of hub HRGs in cell populations. RT-qPCR verified the expression levels of hub HRGs in the human cardiomyocyte model of AMI by oxygen-glucose deprivation (OGD) treatment in AC16 cells.

**Results:**

Fourteen candidate HRGs were identified by differential analysis, and the RF model and the nomogram based on 8 hub HRGs *(IRS2, ZFP36, NFIL3, TNFAIP3, SLC2A3, IER3, MAFF,* and *PLAUR)* were constructed, and the ROC curves verified its good prediction effect in training and validation datasets (AUC = 0.9339 and 0.8141, respectively). In addition, the interaction between hub HRGs and smooth muscle cells, immune cells was elucidated by scRNA analysis. Subsequently, the HRG pattern was constructed by consensus clustering, and the HRG gene pattern verified the accuracy of its grouping. Patients with AMI could be categorized into three HRG subclusters, and cluster A was significantly associated with immune infiltration. The RT-qPCR results showed that the hub HRGs in the OGD group were significantly overexpressed.

**Conclusion:**

A predictive model of AMI based on HRGs was developed and strongly associated with immune cell infiltration. Characterizing patients for hypoxia could help identify populations with specific molecular profiles and provide precise treatment.

## Introduction

Acute myocardial infarction (AMI) is a heart disease in which the local blood supply to the heart is insufficient due to rupture of plaque, aggregation of blood platelets at the rupture site, and formation of a thrombus blocking the coronary artery (Wu X. et al., 2021). Extensive cardiomyocyte death and ventricular remodeling lead to decreased cardiac function, resulting in severe heart failure and increased mortality (Liu et al., 2021). In recent years, advances in interventional cardiology and pharmacologic strategies have significantly improved survival and reduced recurrent ischemic events in patients with AMI (Zhang et al., 2022), as reperfusion therapy and improved antithrombotic treatment have shown promising results in improving cardiac function after AMI (Ibanez et al., 2018). However, the risk of complications in patients with AMI is still high. Precision medicine aims to optimize therapeutic outcomes by tailoring treatment to individual patient characteristics (clinically identified risk factors, biomarkers, pharmacogenomics, etc.) and minimizing adverse events (Sachdeva et al., 2023). It has been shown that optimized antiplatelet therapy based on platelet function assays can provide a personalized treatment approach for patients with acute coronary syndrome (Sotorra-Figuerola et al., 2022). However, risk stratification of patients based on potential risk factors and risk evaluation systems (such as the Global Registry of Acute Coronary Events [GRACE] and the Thrombolysis in Myocardial Infarction [TIMI] risk scores) no longer fully meets the needs of clinical practice (Ma et al., 2021). The combination of traditional models with genetic information, machine learning algorithms, and artificial intelligence techniques can help improve the accuracy of risk assessment. Thus, further exploration of molecular mechanisms and new biomarkers is required.

Persistent ischemia and hypoxia are essential mechanisms in the development of AMI, leading to irreversible myocardial damage and progression of heart failure after AMI (Wu et al., 2019). In a hypoxic environment, a variety of hypoxia-related genes (HRGs) are abnormally expressed during AMI in different regulatory models, such as vascular endothelial growth factor (VEGF), hypoxia-inducible factor (HIF), which are gene products that protect cells from apoptosis and restore blood supply through neovascularization (Kim et al., 2009). Studies have proved the upregulation of the expression of HIF in cardiomyocytes in AMI, activating the hypoxia-related signaling pathway associated with angiogenesis, inflammatory response, and erythropoiesis (Shentu et al., 2020). Duan et al. found that miR-126-3p/TSC1/mTORC1/HIF-1α pathway promotes angiogenesis in myocardial endothelial cells in AMI, improving myocardial function (Duan et al., 2022). It suggests that our exploration of hypoxia-associated signaling pathways will benefit treating AMI.

Areas of myocardial ischemia and hypoxia after AMI recruit and activate immune cells, which produce an inflammatory response in the early stages, contributing to tissue repair and scar formation (Cai et al., 2023). The hypoxic arterial wall lining recruits monocytes via macrophage adhesion ligand (Mac-1), which produces platelet-derived growth factors that induce the proliferation of endothelial cells and smooth muscle cells (SMCs), promoting vascular remodeling and cardiomyocyte repair (Marumo et al., 1999; Schuler et al., 2003). In addition, a study found that hypoxia promotes the expression of M2 macrophage by establishing an AMI mouse model, which is essential in repairing cardiac inflammatory response and fibrosis after AMI (Wang et al., 2023). Neutrophils are attracted by cellular debris, cytokines, and injury-related pattern molecules to accumulate in the region of myocardial infarction, generating highly reactive oxygen species and promoting the secretion of proteases, which can exacerbate tissue and vascular damage (Frangogiannis, 2012). However, recent studies have found that neutrophils promote fibrosis, scar formation, and cardiac remodeling in the injured myocardium by secreting factors that tilt the macrophages toward a segregated phenotype that mediates efficient clearance of cellular debris (Horckmans et al., 2017). These findings suggest that immune cell infiltration is a new direction to prevent the progression of infarction. Exploring the relationship between immune infiltration in infarction and its hypoxia can help analyze the pathogenesis of AMI and provide therapeutic strategies.

**FIGURE 1:**
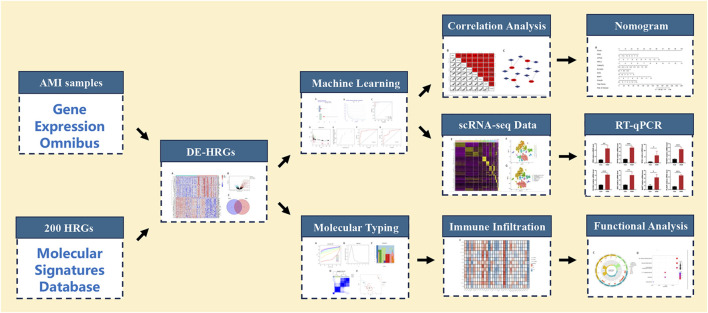
Flowchart for comprehensive analysis of hypoxia-related genes in diagnosis and immune infiltration in acute myocardial infarction. HRGs, hypoxia-related genes; DE-HRGs, differentially expressed HRGs.

In this study, we applied an integrative analysis based on data from bulk RNA and single-cell RNA sequencing (scRNA-seq) to identify the hub HRGs in AMI through machine learning methods, and a diagnostic model of AMI was constructed and validated. The interaction between the immune microenvironment (IME) in AMI and the expression of hub HRGs was also elucidated. The detailed workflow is indicated in Figure 1. A more profound comprehension of the multimolecular characteristics of hypoxia in AMI is anticipated to guide further research by revealing possible biomarkers for diagnosing and treating AMI.

## Material and method

### Data acquisition and processing

We downloaded three datasets from the GEO database (https://www.ncbi.nlm.nih.gov/geo/), the training dataset GSE66360 (49 AMI and 50 normal samples of human), the validation dataset GSE48060 (31 AMI and 21 normal samples of human), and the scRNA dataset GSE163956 (4 AMI and 1 normal samples of mouse). Then, the Perl software was used to add annotation information to the platform file so that the probe matrix could transform into gene expression data. To match gene symbols between different species, we used the “homologene” package for homologous gene conversion. The HRGs were collected from the MSigDB database (Hallmark Gene Set) (https://www.gsea-msigdb.org/gsea/msigdb/).

### Differential expression and correlation analysis

The “limma” package was used to perform differential expression analysis in the control and AMI samples. |log2-fold change (FC)| ≥ 1 and *P* < 0.01 were used as the threshold to identify differentially expressed genes (DEGs). Meanwhile, a heat map of DEGs was plotted using the “pheatmap” package. DEGs were intersected with HRGs to obtain significantly differentially expressed HRGs. A correlation analysis was conducted to understand the interactions between genes, and the gene co-expression network was visualized.

### Construction of machine learning algorithms and nomogram

We used two machine learning algorithms, random forest (RF) and support vector machines (SVM), to identify the hub genes. The RF employs decision trees to evaluate the significance of variables by assigning a value to each one. The SVM is based on nonlinear mapping theory to find the optimal hyperplane of feature space division to help find the key samples and eliminate the redundant samples. The box plots of residuals, inverse distribution plots of residuals, and receiver operating characteristic (ROC) curves were used to assess the algorithms. The RF model was selected based on these results. Candidate genes with a Gini value greater than 2 were further screened, and the “rms” package was employed to construct a nomogram to score the level of each gene to predict the incidence risk of AMI. Calibration curves, decision curve analysis (DCA), clinical impact curve (CIC), and ROC curves were plotted respectively in the training set and testing set to assess the predictive effectiveness of the predictive model.

### Construction of targeted drug and gene interaction network of hub HRGs

The search for targeted drugs of feature genes was based on the Drug-Gene Interaction database (DGIdb) (http://dgidb.genome.wustl.edu/), and the gene-drug network was visualized by the Cytoscpace software (version 3.8.2). The GeneMANIA database (http://genemania.org) was used to explore the functions and interactions of targeted genes.

### Single cell sequencing analysis

The “Seurat” and “SingleR” packages were used to analyze the scRNA-seq data. To retain high-quality data, cells expressing fewer than 300 and more than 30,000 genes, cell numbers less than 3, and mitochondrial gene percentages larger than 10% were all removed. Using the “NormalizeData” function, we normalized the gene expression of the included cells. Then, using the “FindVariableFeatures” function, we extracted the top 20 principal components based on the top 1,500 highly variable genes through principal component analysis (PCA). The cell subpopulations, “FindNeighbors,” “FindClusters” (resolution = 0.4), and “RunTSNE” functions were applied to unsupervised and unbiased clusters. Using the adjusted *P* < 0.01 and absolute log2 (fold change) ≥ 1, the “FindAllMarkers” function screened the marker genes for each cluster. Lastly, the “SingleR” package annotated cell types. The “JackStraw” function visualized and compared the distribution of *P*-values and the uniform distribution of each principal component, and those with significant *P*-values were included in subsequent analyses. Finally, the expression of the hub HRGs in different cells was shown as violin plots.

### Molecular typing and immune infiltration analysis

HRG patterns were identified through the “ConsensusClusterPlus” package based on the expression level of significant differentially expressed HRGs. A PCA algorithm was used to calculate HRG scores for each sample to quantify HRG patterns. PCA was applied to establish a HRG score, namely, the PCA score, and the PCA score was determined based on the formula: PCA score = (PC1*i* + PC2*i*), in which *i* represents the level of HRGs. Both PC1 and PC2 were regarded as signature scores that represent the suspected influence on the sample compositional bias, respectively. Based on the single sample gene set enrichment analysis (ssGSEA), we analyzed the correlation of HRGs with immune cell infiltration and selected HRGs associated with immune infiltration for subsequent studies. The association between differentially expressed HRGs and expression levels of immune checkpoint genes was used to explore possible immunotherapeutic targets.

### Functional enrichment analysis of DEGs

The “limma” package was used to identify and screen the DEGs in HRG mode with |log (FC)| ≥ 1 and *P* < 0.05. Then, we performed gene ontology (GO) and Kyoto Encyclopedia of Genes and Genomes (KEGG) enrichment analysis based on the “clusterProfiler”, “org.Hs.e.g.,.db”, “GOplot”, and “enrichplot” packages, and plotted histograms and bubble plots with the “corrplot” package.

### Cell culture and construction of the model

This study derived the AC16 cells (human cardiomyocyte cells) from the cell bank (Procell; China). We cultured AC16 cells in DMEM (Gibico, Thermo Fisher Scientific, Waltham, MA, United States), which included 10% FBS, 100 U/mL penicillin, and 100 mg/mL streptomycin (Beijing Solarbio Science and Technology Co., Ltd., Beijing, China), at a constant temperature of 37°C and the status of 5% CO_2_.

A suitable density of cells in the logarithmic growth phase was seeded into a medium supplemented with 10% FBS and incubated overnight. AC16 cells were split into the control group (Con group) and the oxygen-glucose deprivation (OGD) treatment group (OGD group).

According to the relevant experimental conditions, the AC16 cells were evenly dispersed and cultured in an incubator for 24 h. We placed the plates in a hypoxic chamber with an inlet and outlet. Next, the outlet port valve was opened, and the anoxic chamber was filled with the mixed gas containing 5% CO_2_ and 95% N_2_ through the inlet port. Shut the air inlet and outlet simultaneously to create an oxygen-free closed chamber, which would be put into an incubator at 37°C. The cells were taken out of the hypoxic chamber after 6 h and needed additional tests by the follow-up experiments.

### The quantitative reverse transcription-polymerase chain reaction (RT-qPCR) analysis

The DMEM solution was supplemented with 10% FBS, where the cells were developed. When the cell confluence rate reached 70%–80%, we cultivated all cells in an incubator with a constant temperature of 37°C and the status of 5% CO_2_. Trypsin solution was employed to digest and subculture these cells, and then cells in the logarithmic growth stage would be removed for further RT-qPCR.

The RNA in each group was extracted by TRIzol kit reagents (Invitrogen, United States) and was reverse transcribed into cDNA by QuanTiect reverse transcription kit (Qiagen, Germany). RT-qPCR was performed with CFX96 Real-time PCR system (Bio-Rad Laboratories, Hercules, CA, United States). *GAPDH* was used to normalize expression levels. Table 1 contains a list of primer sequences. The 2^−ΔΔCT^ approach was used for data analysis.

**TABLE 1:** Primers used for RT-qPCR.

Gene	Primers	Sequence (5′–3′)
*IRS2*	Forward	ACACCTACGCCAGCATTGAC
	Reverse	GCCTTGTTGGTGCCTCATCT
*ZFP36*	Forward	GGGAGGCAATGAACCCTCTC
	Reverse	GCAACGGCTTTGGCTACTTG
*NFIL3*	Forward	GGAGCCAAGAGATGACCGAG
	Reverse	TGGAGGATCGGTTGACTTGC
*TNFAIP3*	Forward	ATCCGAGCTGTTCCACTTGTTAA
	Reverse	CAACTTTGCGGCATTGATGAGAT
*SLC2A3*	Forward	CACGCTCATGACTGTTTCTTTGT
	Reverse	CTGAAGAGTTCGGCCACAATAAA
*IER3*	Forward	GCCGCCTTCTAACTGTGACT
	Reverse	CGTCTCCGCTGTAGTGTTCT
*MAFF*	Forward	GAGCTGAGCGAGAACACG
	Reverse	GTAGCCACGGTTTTTGAGTGT
*PLAUR*	Forward	ATGCATTTCCTGTGGCTCATCAG
	Reverse	GAAGGTGTCGTTGTTGTGGAAAC
*GAPDH*	Forward	GGAGTCCACTGGCGTCTTCA
	Reverse	GTCATGAGTCCTTCCACGATACC

### Statistical analysis

This study processed and analyzed data by Perl software (version 5.18.2) and R software (version 4.1.2). Student's t-test or Wilcoxon's rank sum test was used to detect the significant difference between the two independent groups. *P* < 0.05 was generally considered statistically significant.

## Result

### Identification of differentially expressed HRGs

**FIGURE 2:**
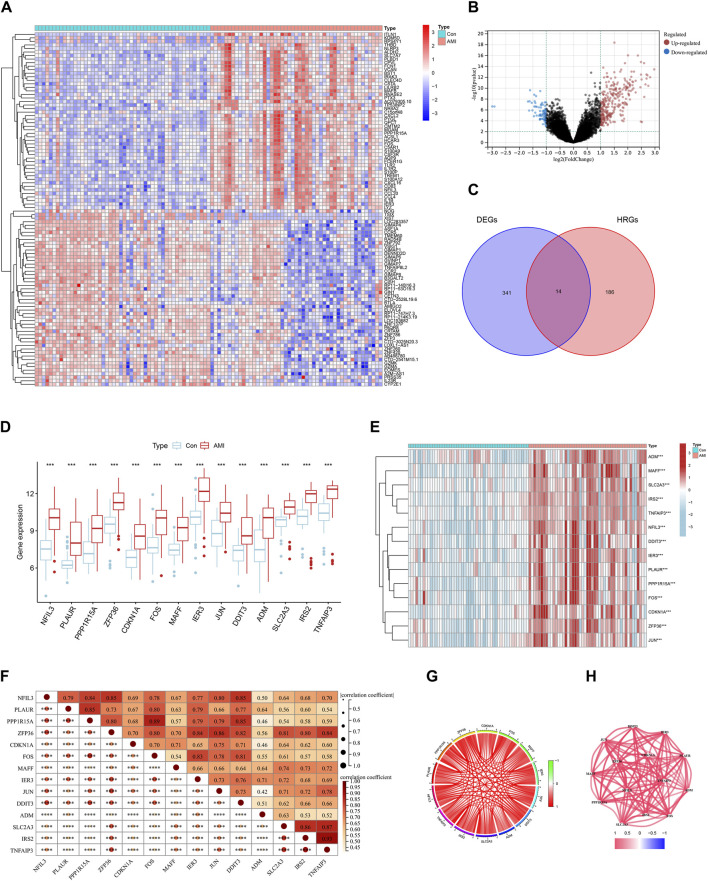
Identification of differentially expressed hypoxia-related genes (HRGs) in acute myocardial infarction (AMI). **(A)** Heat map of differentially expressed genes (DEGs); **(B)** Volcano plot of DEGs; **(C)** Venn diagram for the intersection of DEGs and HRGs; **(D)** Boxplots for 14 candidate genes between control and AMI groups; **(E)** Expression heatmap of 14 candidate genes; **(F)** Correlation heat map of 14 candidate genes; **(G)** Chordal graph of 14 candidate genes correlations of 14 candidate genes; **(H)** Interaction network of 14 candidate genes. ^***^ *P* < 0.001; ^****^ *P* < 0.0001.

We obtained 355 DEGs by differential analysis between healthy individuals and patients with AMI. The heatmap showed the top 50 genes with significant up- or downregulated expression (Figure 2A). The volcano map indicated the overall distribution of expression levels and fold change of DEGs (Figure 2B). We took the intersection of the obtained DEGs with 200 HRGs and finally screened out 14 candidate genes (*NFIL3, PLAUR, PPP1R15A, ZFP36, CDKN1A, FOS, MAFF, IER3, JUN, DDIT3, ADM, SLC2A3, IRS2,* and *TNFAIP3*) (Figure 2C). These candidate genes were consistently highly expressed in AMI and may play an important role (Figures 2D,E). Correlation analysis was carried out to understand the association between candidate genes better. It showed a strong positive correlation between each gene (Figures 2F,G). Figure 2H shows the correlation networks of 14 candidate genes with a correlation coefficient > 0.4, reflecting the strong correlation between these genes.

### Selection and validation algorithm

**FIGURE 3:**
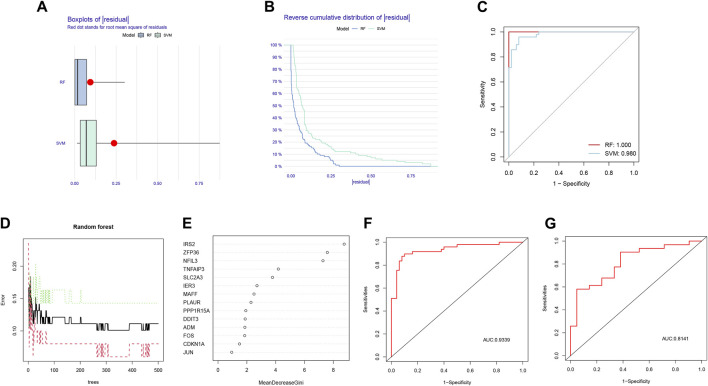
Comparison and selection of random forest (RF) and support vector machines (SVM). **(A)** Residual Boxplot of RF and SVM, where the red dots indicate the root mean square of the residuals; **(B)** Reverse cumulative distribution of residual; **(C)** Receiver operating characteristic (ROC) curve of RF and SVM. The AUC of RF and SVM were 1.000 and 0.980, respectively; **(D)** RF prediction error curves based on 10-fold cross-validation curve; **(E)** The scoring plot of each gene; **(F)** The ROC curve of the logistic regression model constructed in acute myocardial infarction (AMI) based on hub HRGs identified by RF algorithm in the training cohort; **(G)** The ROC curve of the logistic regression model constructed in AMI in the testing cohort.

Based on the comparison between RF and SVM algorithms, we found that the RF algorithm had lower residuals than the SVM algorithm, suggesting it was more accurate for forecasting the risk of AMI (Figures 3A, B). Furthermore, the ROC curve was plotted to compare the effect of the two approaches (Figure 3C). The finding that the AUC of RF was higher than that of SVM further illustrated that the former was the more appropriate algorithm.

According to the prediction error curves of the RF algorithm based on a 10-fold cross-validation curve, the best “ntree” was selected (Figure 3D). Figure 3E represented the importance score of each gene; 8 hub HRGs, including *IRS2, ZFP36, NFIL3, TNFAIP3, SLC2A3, IER3, MAFF,* and *PLAUR*, were screened based on the criterion of Gini value greater than 2.

Based on the 8 hub HRGs, a binary logistic regression model was used to predict the risk of AMI. The ROC curves were exhibited in Figures 3F, G. The discrimination power of the model achieved good performance in both training and testing sets (AUC = 0.9339 and 0.8141, respectively). The above findings revealed that the screened hub HRGs based on the RF algorithm could be implemented as a valid model to predict the occurrence of AMI.

### Establishment of the nomogram

**FIGURE 4:**
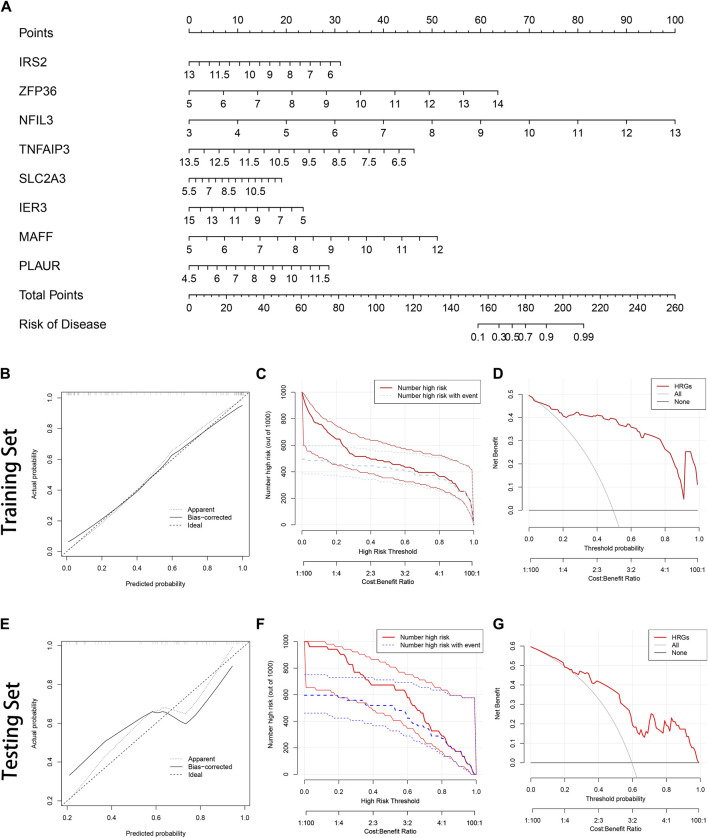
Construction of the nomogram. **(A)** The nomogram is based on 8 hub hypoxia-related genes (HRGs). The expression level of each HRG in the graph corresponds to the score of the upper score scale, and the researchers can read out the corresponding score of each HRG according to the actual situation and get the total score by summing it up. Ultimately, the total score can be added to the probability of AMI risk at the bottom of the graph; **(B)** The calibration curve in the training set; **(C)** The clinical impact curve (CIC) in the training set; **(D)** Decision curve analysis (DCA) in the training set; **(E)** The calibration curve in the testing set; **(F)** The CIC in the testing set; **(G)** The DCA in the testing set.

Each gene was scored, and the risk of AMI was predicted based on the sum; the specific way of assigning points was revealed in the nomogram (Figure 4A). The bias-corrected line was close to the ideal line (Figure 4B), illustrating the model’s good prediction ability. Besides, DCA was performed in which the curve of HRGs deviated from two extreme ones, indicating the model’s potential clinical utility (Figure 4C). The CIC proved the availability of a nomogram by comparing the number of predicted and actual patients under different probability thresholds (Figure 4D). In addition, we further evaluated the modeling effects in the testing set (Figures 4E–G). It was consistent with the training set, reflecting that the model had better generalization ability.

### Relationship among 8 hub HRGs and target drugs

**FIGURE 5:**
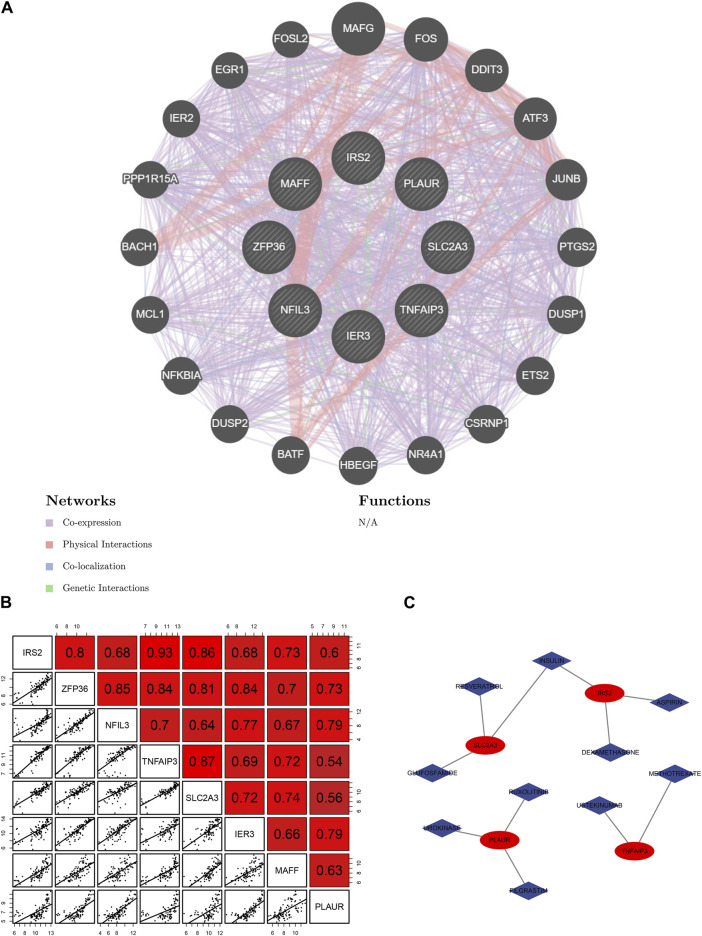
Relationship among 8 hub hypoxia-related genes (HRGs) and target drugs. **(A)** Interaction networks of 8 hub HRGs based on the geneMANIA database; **(B)** Correlation analysis among 8 hub HRGs; **(C)** Gene-drug regulatory network.

The network demonstrated the connection between the genes based on the geneMANIA (Figure 5A). *NFIL3* had stronger physical interactions with *MAFF*, *MAFG, DDIT3,* and *BATF.* Genetic interaction existed between *IRS2 and NFIL3, IER3,* and *TNFAIP3*. The genes had a specific positive correlation, and the correlation between *IRS2* and *TNFAIP3* was the highest (Figure 5B). Targeted drugs against *SLC2A3, IRS2, PLAUR,* and *TNFAIP* are shown in Figure 5C. Insulin was a targeted drug common to *SLC2A3* and *IRS2*.

### Single-cell analysis indicated the hub HRGs interact with the SMCs and immune in AMI

**FIGURE 6:**
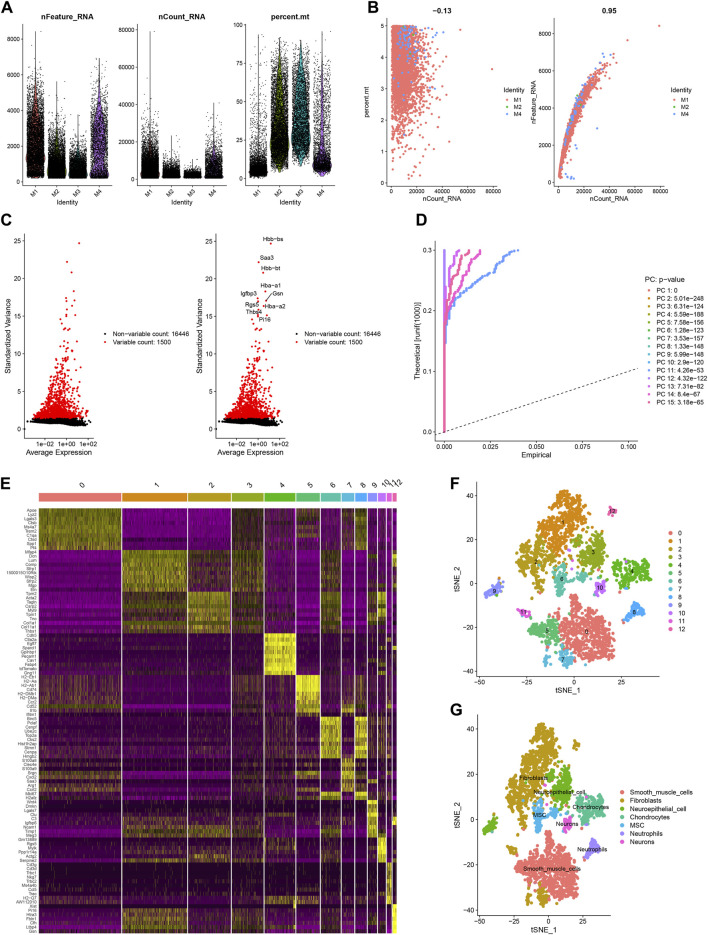
Sample quality control and cell annotation. **(A)** Violin plots of quality control metrics: The gene counts per cell (nCandidate_RNA), number of unique molecular identifiers (UMIs) per cell (nCount_RNA), and percentage of mitochondrial genes per cell (percent. mt); **(B)** Scatterplots with different modes of quality control; **(C)** The variance plot: red dots revealed 1,500 highly variable genes; **(D)** JackStraw Plot; **(E)** DimHeatmap; **(F)** Visualization results of t-SNE dimensionality reduction clustering; **(G)** 7 cell types were annotated.

**FIGURE 7:**
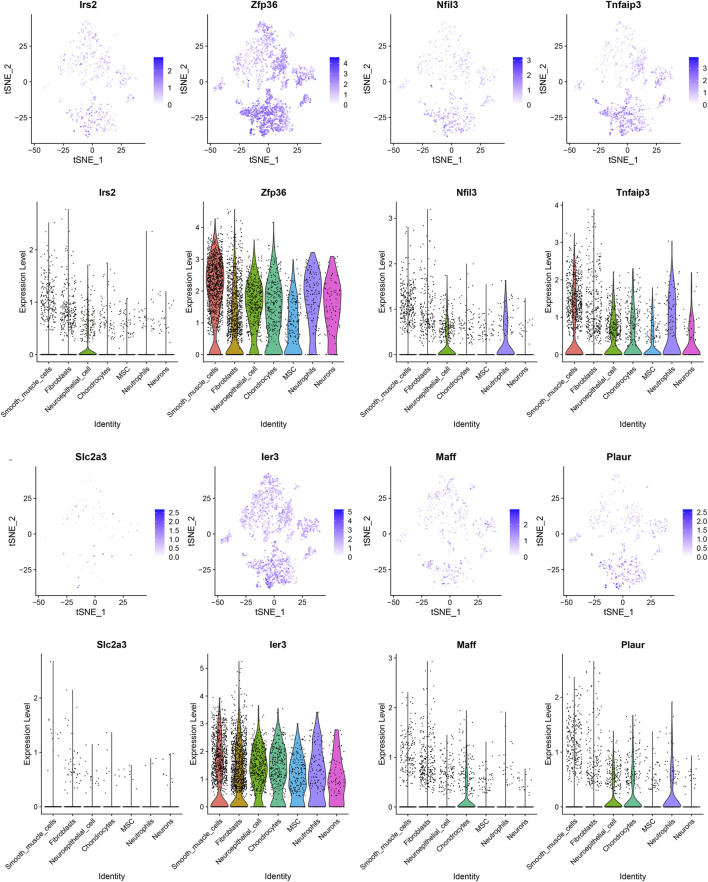
The t-SNE plot based on the expression of 8 hub hypoxia-related genes and violin plots of gene expression in different cells.

We filtered ineligible cells according to quality control criteria (Figure 6A). The nCount RNA positively correlated with nFeature RNA, with a correlation coefficient of 0.95 (Figure 6B). As shown in Figure 6C, 1,500 highly variable genes were screened. The “RunPCA” function performed dimensionality reduction, and 13 clusters in total were recognized (Figures 6D, F). The heatmap exhibited the top 10 marker genes in each cluster (Figure 6E). At last, the “SingleR” function was employed to annotate, and 7 cell types, containing SMCs, fibroblasts, neuroepithelial cells, chondrocytes, MSCs, neutrophils, and neurons, were visualized in different colors (Figure 6G). The distribution of 8 hub HRGs in different kinds of cells was displayed in Figure 7. We noticed that *Zfp36, Tnfaip3,* and *Ier3* were distributed in various cells, among which SMCs expressed more. Besides, these 6 genes (except *SIc2a3* and *Maff*) were somewhat expressed in neuroepithelial cells. Expression of *Zfp36, Tnfaip3, Ier3,* and *Plaur* may be associated with neutrophils.

### Identification of three HRG patterns

**FIGURE 8:**
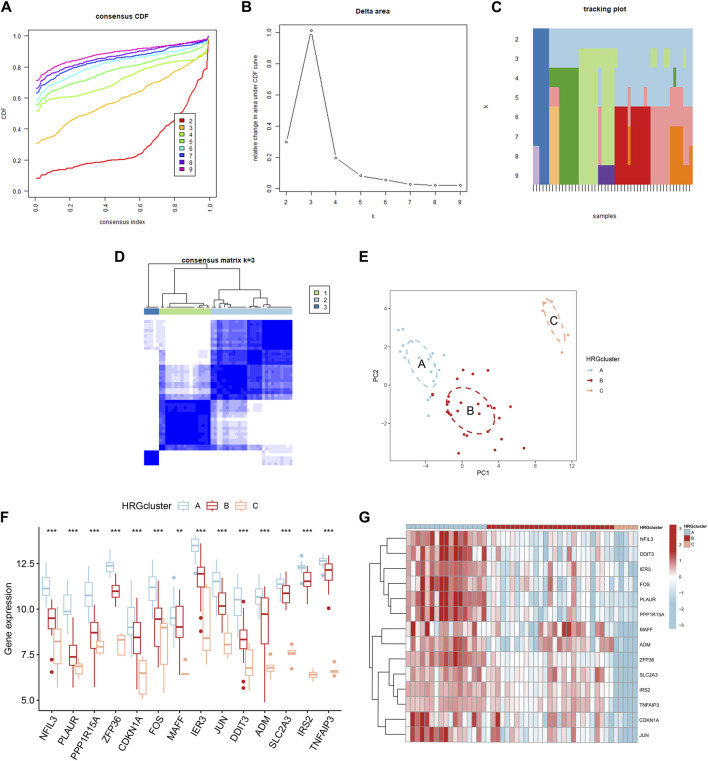
Consensus clustering of 14 differentially expressed hypoxia-related genes (HRGs) in patients with acute myocardial infarction. **(A)** Cumulative distribution function (CDF); **(B)** Delta area plot of consensus clustering; **(C)** Tracking plot of 14 differentially expressed HRGs; **(D)** Consensus matrix with k = 3; **(E)** Principal component analysis (PCA); **(F)** Expression boxplots of 14 differentially expressed HRGs in different clusters; **(G)** Heatmap of expression of 14 differentially expressed HRGs in different clusters. ^**^ *P* < 0.01; ^***^ *P* < 0.001.

Based on the 14 differentially expressed HRGs and the results of Figures 8A–D, we chose k = 3 for unsupervised consensus clustering. According to the results of PCA, the AMI samples were effectively distinguished 3 HRG patterns (Figure 8E). The HRG clusters were divided into 3 subclusters for differential analysis, and significant heterogeneity in the expression of the 14 differentially expressed HRGs in the three clusters was observed, with the expression of the genes in cluster A being significantly higher than those in clusters B and C (Figures 8F,G).

### Immune infiltration analysis

**FIGURE 9:**
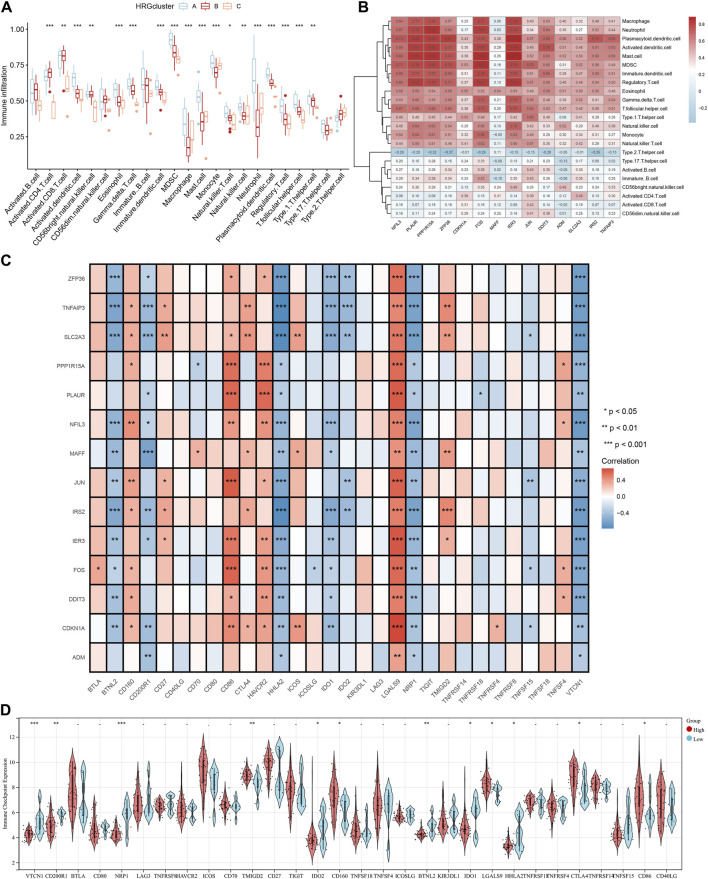
Immune cell infiltration analysis. **(A)** The correlation between infiltrating immune cells and HRG clusters; **(B)** Heatmap between 14 differentially expressed hypoxia-related genes (HRGs) and the expression level of immune cell in different clusters; **(C)** The correlation analysis between 14 differentially expressed HRGs and immune checkpoint; **(D)** Violin plot of the immune checkpoint in high and low HRG scores groups. ^*^ *P* < 0.05; ^**^ *P* < 0.01; ^***^ *P* < 0.001.

Immune infiltration analysis was performed by ssGSEA to investigate the relationship between different subpopulations and immune cell infiltration. The results showed that the 14 differentially expressed HRGs were significantly positively correlated with a variety of immune cells, including macrophages, neutrophils, and regulatory T cells (Figures 9A, B). In the immune checkpoint analysis, all 14 differentially expressed HRGs showed a significant positive correlation with *LGALS9* and a significant negative correlation with *HHLA2, NRP1,* and *VTCN2* (Figure 9C). To further investigate the expression differences at the immune infiltration sites, we divided AMI samples into high and low groups of HRG scores. It showed that the expression of *VTCN1, CD200R1, NRP1,* and *BTNL2* was downregulated in the high-expression group of HRG scores. In contrast, the expression of *TMIGD2* was upregulated (Figure 9D).

### Identification of three HRG gene patterns

**FIGURE 10:**
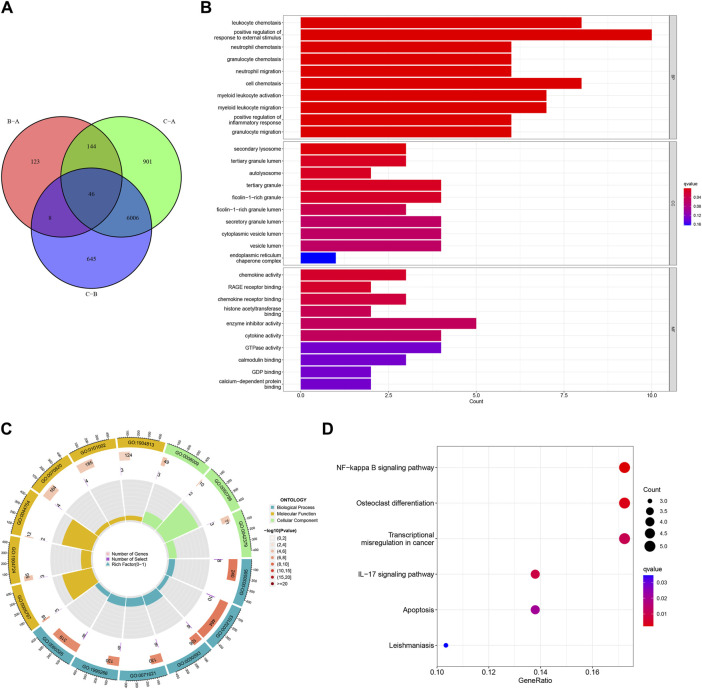
Functional enrichment analysis based on the hypoxia-related gene (HRG) patterns. **(A)** Venn diagram of interactions between different clusters; **(B)** Bar chart for gene ontology (GO) analysis, the redder the color, the more significant the enrichment of the genes in the pathway; **(C)** The GO Circle Chart includes the analysis of the biological process, molecular function, cellular component; **(D)** A bubble chart for Kyoto Encyclopedia of Genes and Genomes (KEGG) analysis.

Forty-six DEGs were screened under three different HRG patterns by the “limma” package (Figure 10A). GO and KEGG analyses were performed to explore the potential pathways of DEGs in AMI. The GO results showed that DEGs were mainly enriched in leukocyte chemotaxis, neutrophil chemotaxis, neutrophil migration, granulocyte neutrophil, and positive regulation of inflammatory response in the biological process. Secondary lysosome, tertiary granule lumen, and ficolin-1-rich granule were significantly enriched for cellular components. In addition, DEGs were mainly enriched in chemokine activity and chemokine receptor binding in the molecular function (Figures 10B, C). In KEGG analysis, DEGs were mainly enriched in the NF-kappa B signaling pathway, IL-7 signaling pathway, and apoptosis (Figure 10D).

**FIGURE 11:**
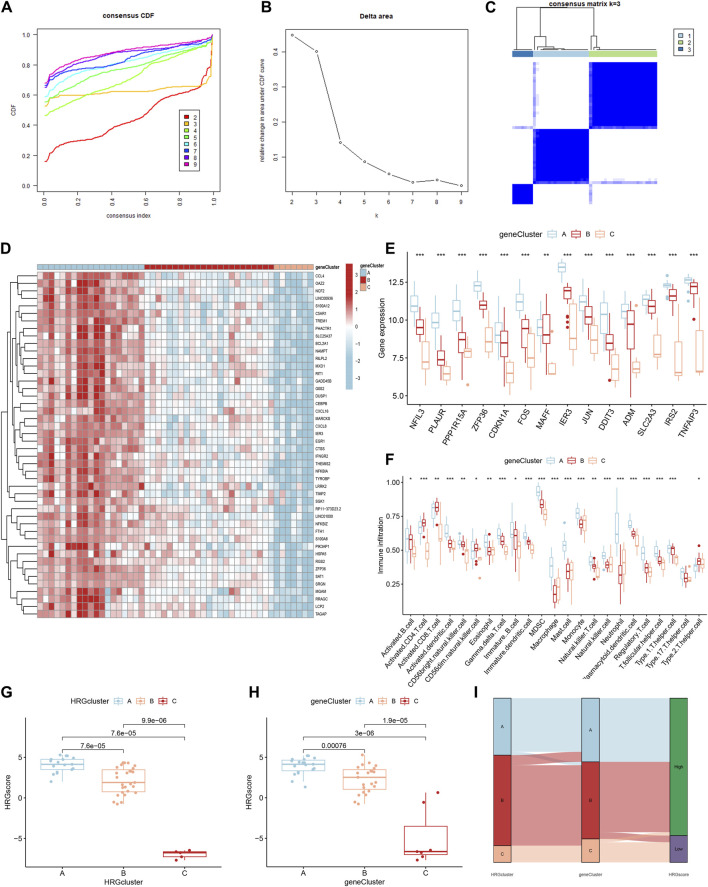
Identification of gene patterns related to hypoxia-related genes (HRGs). **(A)** Cumulative distribution function (CDF); **(B)** Delta area plot of consensus clustering; **(C)** Consensus matrix with k = 3; **(D)** Heatmap of HRGs with immune checkpoint profiles among the gene clusters; **(E)** Gene expression in three clusters; **(F)** The correlation analysis between 14 differentially expressed HRGs and immune infiltrating; **(G)** The score of HRG clusters; **(H)** The score of gene clusters; **(I)** The Sankey diagram showed the relationship between HRG clusters, gene clusters, and HRG scores. ^*^ *P* < 0.05; ^**^ *P* < 0.01; ^***^ *P* < 0.001.

To further validate the HRG pattern, patients with AMI were again categorized into 3 different gene patterns by unsupervised consensus clustering, and the results were consistent with the HRG pattern (Figures 11A–C). The expression levels of the 14 differentially expressed HRGs with immune cell infiltration among the gene clusters were similar to the results of the HRG pattern (Figures 11D–F). In addition, we also compared the HRG scores between different HRG clusters and HRG gene clusters, and the results showed that the HRG cluster A or gene cluster A had the highest score, and the HRG cluster C or gene cluster C had the lowest score (Figures 11G, H). The Sankey diagram showed the relationship between HRG clusters, gene clusters, and HRG scores. (Figure 11I).

### Expression of hub HRGs analyzed by RT-qPCR

**FIGURE 12:**
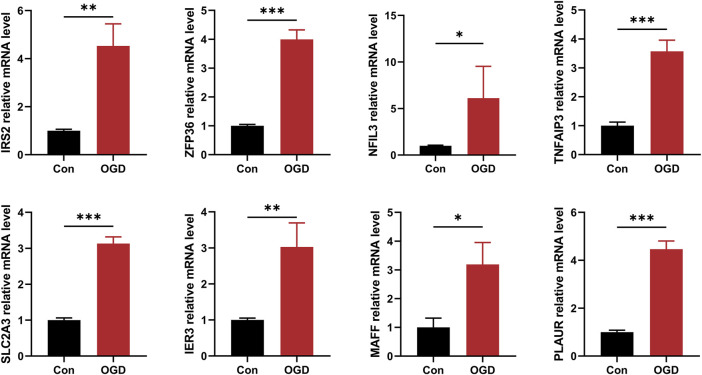
The quantitative reverse transcription-polymerase chain reaction (RT-qPCR) to detect expression levels of 8 hub hypoxia-related genes. ^*^ *P* < 0.05; ^**^ *P* < 0.01; ^***^ *P* < 0.001.

By treating AC16 cells with oxygen and glucose deprivation, we constructed a cellular model of AMI to further validate the correctness of our results. The expression level of 8 hub HRGs, *IRS2, ZFP36, NFIL3, TNFAIP3, SLC2A3, IER3, MAFF,* and *PLAUR*, was significantly increased in the OGD group (Figure 12). Consistent with the results of bioinformatics analysis, the expression of hub HRGs was upregulated in AC16 cells with OGD treatment.

## Discussion

Hypoxia is a typical pathologic process in various development mechanisms in AMI. In this study, we identified 8 hub HRGs *(IRS2, ZFP36, NFIL3, TNFAIP3, SLC2A3, IER3, MAFF,* and *PLAUR)* and constructed a diagnostic model to predict the occurrence of AMI. Here, we applied a comprehensive analysis of bulk RNA-seq and scRNA-seq data and found that the identified hub HRGs were closely associated with the IME of AMI.

Inadequate oxygen supply to the heart triggers a series of responses, including mitochondrial degradation, free radical release, inflammatory cell aggregation, lactic acidosis, and angiogenesis. HIF is a vital transcription factor with a broad spectrum of target genes, which is stably expressed during hypoxia and drives the initial response to hypoxia (Knutson et al., 2021). Promoting cardiomyocyte proliferation and repair under hypoxia is a therapeutic strategy for AMI, and studies have shown that increased glucose metabolism favors the regeneration of neonatal mouse hearts (Zhu et al., 2022). IRS2 regulates the transduction of insulin and insulin growth factor, and research has demonstrated that the HIF-2α/IRS2 pathway can connect hypoxia sensing to the inhibition of gluconeogenesis (Wei et al., 2013). Additionally, IRS2 has anti-inflammatory properties in the hypoxic microenvironment and can limit macrophage activation (Nakahara et al., 2021). SLC2A3 and GLUT3 are involved in the transmembrane transport of glucose and affect the transformation of CD4^+^ T-cell to regulate inflammation (Yang et al., 2023). HIF-1 promoted glycolysis during hypoxia, and SLC2A3 was one of its target genes, promoting glucose uptake and utilization. The HIF1/SLC2A3 axis is closely related to endothelial function and may facilitate HIF pro-angiogenesis (Mimura et al., 2012). The expression of ZFP36 is elevated in coronary epithelial cells of injured human hearts, which can regulate their proliferation and maybe a new target for vascular regeneration (Li et al., 2023). ZFP36 is a zinc finger protein, and Wu et al. found that ZFP36L2 can attenuate mitochondrial fusion and fission by affecting PVT1, thus alleviating myocardial injury (Wu F. et al., 2021). Meanwhile, the zinc finger structural domain can bind to the untranslated region of mRNA to interfere with post-transcriptional modification and translation, reducing the expression of inflammatory cytokines.

During AMI, many inflammatory cells infiltrate into the myocardium, starting with rapid neutrophil aggregation, followed by monocyte and macrophage infiltration. Then, fibroblasts are activated and recruited to participate in the repair process (Frangogiannis, 2014; Zhu et al., 2022). It has been reported that NLRP3 inflammasome activation in immune cells recruited to the foci of myocardial injury leads to further myocardial injury. TNFAIP3 has been confirmed to inhibit inflammasome activation in bone marrow-derived macrophages. TNFAIP3 is a dual ubiquitin-modifying enzyme with anti-inflammatory and inhibitory NF-κB effects (Zhang et al., 2018). Giral et al. discovered that the TNFAIP3 levels in the blood mononuclear cells of patients with AMI were positively correlated with the degree of cardiac damage and were thought to be associated with attenuated inflammasome activation (Giral et al., 2022). TNFAIP3 may exert anti-inflammatory effects by blocking NF-κB and its dependent proteins and affecting the signaling to negatively regulate the activation of inflammasomes in macrophages by toll-like receptors (Patel et al., 2006; Yuk et al., 2015). PLAUR is a multidomain glycoprotein-activated receptor that binds PLAU and further activates fibrinogen to degrade extracellular matrix to promote cell metastasis; on the other hand, it can form complexes with vitronectin and integrins to enhance monocyte adhesion (Bai et al., 2021). Besides, it can also secrete cytokines to activate the PI3K/Akt signaling pathway to trigger inflammation.

The prediction model constructed based on hub HRGs achieved satisfactory results in both training and testing sets. The scRNA-seq analysis was completed to investigate the relationship between each gene and different cells, identifying 8 core cells, among which SMCs attracted our attention. SMCs are closely associated with the arterial remodeling process after vascular injury, which may improve the therapeutic strategy for AMI (Shi et al., 2019). Zinc deficiency has been reported to be a promoter of endothelial atherosclerosis. Zinc supplementation can exert an inhibitory effect on vascular calcification by suppressing NF-κB through upregulation of TNFAIP3 (Voelkl et al., 2018). In addition, TNFAIP3 can inhibit the proliferation of SMCs to prevent endothelial hyperplasia without causing apoptosis and increase the sensitivity of SMCs to apoptosis in *de novo* endothelial lesions, which may be necessary for treating endothelial diseases (Patel et al., 2006). IER3 is an NF-κB-responsive gene similar to TNFAIP3, which is also upregulated in atherosclerosis, inhibited endothelial neogenesis, and has an anti-myocardial hypertrophic function. It has been revealed that biomechanical stimulation under the transcriptional control of NF-κB activation induced IER3, and adenoviral gene transfer of IER3 had an obvious inhibitory effect on the proliferation of SMCs *in vitro* (Schulze et al., 2003). A study by Brahmbhatt et al. also illustrated that IER3 gene deletion inhibited venous neointimal hyperplasia (Brahmbhatt et al., 2014). The high expression of these genes in SMCs may reflect the negative feedback regulation mechanism after vascular injury in AMI, providing new ideas for diagnosing and treating the disease.

This study classified AMI samples into 3 HRG subclusters based on 14 differentially expressed HRGs and analyzed them for immune infiltration. The expression of each gene decreased sequentially in clusters A, B, and C. Most of the significantly different immune cell infiltrations displayed the same trend. However, macrophages, mast cells, monocytes, and neutrophils were more frequently distributed in cluster C than in cluster B. Moreover, we analyzed immune checkpoints to provide new insights into immunotherapy for AMI. LGALS9 was an immune checkpoint that significantly positively correlated with all 14 differentially expressed HRGs. As early as 2015, a study demonstrated that serum LGALS9 was associated with the degree of coronary artery stenosis and inhibited the development of atherosclerosis in Chinese (Zhu et al., 2015). In a study on AIDS, LGALS9 levels were found to predict the probability of myocardial infarction or stroke after receiving antiretroviral therapy (Premeaux et al., 2021). HHLA2, a B7 family ligand, is highly expressed in various cancers, is associated with immune evasion, and has a bi-directional regulatory effect on T cells. Meanwhile, it correlates with hypoxia in the tumor microenvironment, but the association needs further study (Mortezaee, 2023). Upregulation of each gene blocks HHLA signaling and may have therapeutic effects by improving T-cell proliferation.

We performed functional enrichment analysis to further explore the possible biological functions played by the DEGs. GO analysis exhibited significant enrichment of chemotaxis and migration of multiple immune cells, including neutrophils, leukocytes, myeloid leukocytes, and granulocytes. It reflected the vital role of the inflammatory response in myocardial injury and repair. KEGG analysis showed aggregation in the NF-κB signaling pathway. The NF-κB signaling pathway was redox-sensitive, and during myocardial ischemia and hypoxia, HIF-α was translocated to neutrophil nuclei, and the NF-κB pathway was activated, mediating cytokine transcription. The TLR4/MyD88/NF-κB pathway is an important pathway that ameliorates inflammatory injury disease, attenuating apoptosis after AMI (Zhang et al., 2022). NF-κB was regulated by various miRNA. Huang et al. found that IL-1β promotes the proliferation of hypoxic vascular endothelial cells through miR-24-3p/NKAP/NF-κB axis (Huang et al., 2022). The research written by He et al. illustrated that dexmedetomidine can exert cardioprotective effects by inhibiting the NF-κB pathway through miR-1a-6p targeting IRAK146 and TRAF3 (He et al., 2021).

In the current study, we comprehensively analyzed the specific role of HRGs in the diagnosis and immune infiltration of AMI based on bulk and single-cell RNA sequencing data, which provides new perspectives for subsequent studies. However, this study still had some limitations. First, data were obtained from only one database, and the limited sample size may have affected the statistical analysis results to some extent. Second, studies with larger samples are needed to further evaluate the risk prediction model. Third, the assessment of HRGs was limited to the RNA level only; refinement of multi-omics studies such as proteomics and metabolomics was expected. Fourth, although we normalized and filtered the data, there was still a batch effect due to the use of different platforms and different dates of processing and analysis. The batch effect will be more obvious, especially when analyzing different data from various sequencing studies. A large amount of genetic data is still needed to validate our conclusions in the future. Finally, inflammation and immune cell infiltration into the infarcted area are essential manifestations of the hypoxic features. Correlation analyses do not allow an account of their specific roles; more in-depth studies are needed to validate our conclusion.

## Conclusion

In this study, we utilized a machine learning approach to select 14 candidate HRGs and finalized 8 hub HRGs *(IRS2, ZFP36, NFIL3, TNFAIP3, SLC2A3, IER3, MAFF,* and *PLAUR)* to be used to construct a risk prediction model for AMI, which demonstrates the potential of hub HRGs as biological markers for AMI. The scRNA-seq analysis further revealed the important role of SMCs and immune cells in AMI. HRGs and gene subclusters were identified by consensus clustering, and their relationship with immune cell infiltration was further explored. Based on these findings, characterizing patients for hypoxia could help identify populations with specific molecular profiles and provide precise treatment. However, more multi-omics studies are still needed to further explore the hypoxic features in AMI and provide new strategies for diagnosis, classification, targeted therapy, and prognosis prediction.

## Data Availability

The original contributions presented in the study are included in the article/supplementary material, further inquiries can be directed to the corresponding author.
